# A randomized controlled trial of the Happy, Healthy, Loved personalized text-message program for new parent couples: impact on breastfeeding self-efficacy and mood

**DOI:** 10.1186/s12884-024-06684-9

**Published:** 2024-07-26

**Authors:** Erin Henshaw, Marie Cooper, Teresa Wood, Sanchita Krishna, Marie Lockhart, Stacey Doan

**Affiliations:** 1 Department of Psychology, Denison University, 100 W. College St, Granville, OH 43023 USA; 2 Riverside Methodist Hospital, OhioHealth, 3535 Olentangy River Rd., Columbus, OH USA; 3 OhioHealth, 3535 Olentangy River Rd, Columbus, OH 43214 USA; 4 OhioHealth Research Institute, 3535 Olentangy River Rd, Columbus, OH 43214 USA; 5 Berger Institute, Claremont McKenna College, 888 N. Columbia Ave, Claremont, CA 91711 USA

**Keywords:** Breastfeeding, Breastfeeding self-efficacy, Postpartum depression, Fathers, Social support, Couples, Intervention, Psychosocial, Text-message, Coping, Mental health

## Abstract

**Background:**

Breastfeeding self-efficacy has been identified as an important influence on breastfeeding outcomes. Among new parent couples, partners are uniquely positioned to be sources of support for developing breastfeeding self-efficacy, yet few breastfeeding programs have attempted to involve partners directly. The purpose of this study was to test the impact of a novel program, *Happy, Healthy, Loved*, on breastfeeding self-efficacy and maternal mood through emphasizing partner support and actively addressing postpartum-specific stress management in a tailored text message delivery program.

**Methods:**

A randomized trial was conducted in which primiparous mother-partner dyads intending to exclusively breastfeed were recruited at midwestern hospitals 2–3 days after delivery. The clinical trial was pre-registered at clinicaltrials.gov (#NCT04578925, registration date 7/24/2020). Couples were randomized to receive intervention or an attentional control. Couples randomized to the intervention group then completed a brief interactive educational tablet program together (Happy, Healthy, Loved), followed by 6 weeks of tailored text messages providing reminders, coping strategies, and motivational milestones to improve breastfeeding self-efficacy. Participants in the control group received usual care followed by 6 weeks of attentional control text messages about infant development. Surveys were delivered at baseline, 6 weeks, and 6 months postpartum to both mother and partner to assess breastfeeding self-efficacy, mood, and social support (*n* = 62 couples).

**Results:**

Outcomes of ANCOVA with baseline self-efficacy as a covariate showed a significant effect of intervention on 6 months breastfeeding self-efficacy when compared to control group. No other significant differences were found at 6 weeks or 6 months postpartum in breastfeeding self-efficacy, depressive or anxious symptoms.

**Conclusions:**

Results of the present investigation suggest that a text-based dyad intervention improved breastfeeding self-efficacy at 6 months, but not 6 weeks, postpartum, indicating that text-based mother-partner interventions are a promising direction to continue exploring in postpartum health research.

**Trial registration:**

Clinicaltrials.gov #NCT04578925.

## Background

Breastfeeding’s health benefits are extensive and well established, yet exclusive breastfeeding rates remain low in the United States [1–7]. For infants, health risks such as sudden infant death syndrome, infections, diabetes, obesity, and asthma are lower [1, 3]; for mothers, breastfeeding is associated with lower risks of breast and ovarian cancers [4]. For this reason, major health organizations, including the World Health Organization, recommend exclusive breastfeeding for at least the first 6 months postpartum [3, 5, 6]. However, exclusive breastfeeding in the US is reported by only 44.4% of women at 3 months postpartum [7]. Therefore, improving exclusive breastfeeding rates is among the nation’s health priorities outlined in Healthy People 2020 [7].

While many factors may contribute to early weaning, data suggest that, for many mothers, weaning is often involuntary [8, 9]. One of the most widely recognized risk factor for involuntary weaning is the lack of breastfeeding self-efficacy (BSE) [10]. Self-efficacy, or confidence in one’s ability to breastfeed, has been found to predict effort and perseverance through common challenges such as early latching difficulties, perceived supply concerns, and return to work [11–13]. The development of BSE is instantiated by four key factors: performance accomplishments in breastfeeding, exposure to other women’s breastfeeding experience, encouragement from influential others (such as partners), and physiological responses such as stress, anxiety, and fatigue [10].

Importantly, evidence suggests that BSE is a modifiable factor [14]. Two systematic reviews found that BSE interventions effectively improve exclusive breastfeeding duration; however, most interventions are delivered in one-on-one interventions with multiple contact points, which can be cost and time intensive [14, 15]. Moreover, these interventions are time limited, unable to provide dynamic support that matches the changing needs and challenges of breastfeeding across time. Mobile phone text message delivery, which has been found to be efficacious in altering other health behaviors [16], offers a cost-effective direction for increasing points of contact in future BSE interventions.

In addition to technical and instructional support for breastfeeding, the BSE framework emphasizes the positive management of stress and physiological arousal as a technique to increase breastfeeding duration. The relationship of negative emotional states or stress and breastfeeding has been well established [17, 18] and lower BSE has been associated with less effective emotion regulation strategies [19]. Neuroendocrine activity associated with reported stress and anxiety may interfere with production and letdown of milk [9, 20], while cognitive patterns such as rigid expectations and focus on self-deficiencies may result in less rewarding breastfeeding experiences, ultimately contributing to avoidance and discontinuation [21].

Effective approaches to managing stress and arousal include engaging in constructive internal dialogue and managing emotional reactions to challenges along with enhanced ability to produce calm, relaxed physiological states [22]. In the perinatal period, cognitive-behavioral theory is the basis of several efficacious programs to produce positive coping for the prevention of stress and negative emotional states, though not exclusively focused on breastfeeding outcomes [22–24]. Core components include cognitive strategies to foster flexible, self-compassionate, realistic interpretation of one’s self and environment, coupled with behavioral strategies to increase rewarding or meaningful activities, increase support networks, and challenge negative self-beliefs [25]. Previous randomized controlled trials suggest that cognitive behavioral coping skills can be effectively taught via internet and mobile delivery [23, 26–28].

While most breastfeeding interventions focus exclusively on mothers, the inclusion of co-parent partners when applicable has the potential to increase the instrumental and social-emotional benefits of any intervention. Among partnered women,^1^ partners are uniquely positioned to be sources of support and influential in developing BSE during the postpartum period, yet few breastfeeding programs have attempted to involve partners directly. Developing programs involving partners is a recommended action in the Surgeon General’s report on breastfeeding, due to the important influential role of the partner [29]. Fathers’ support of breastfeeding is associated with higher rates of breastfeeding initiation, duration, and exclusivity [30, 31]. Research suggests that fathers feel willing but unprepared to support breastfeeding [32] suggesting a need for interventions directly equipping them for this role.

### Current study

The primary aim of the current study is to test the efficacy of a mobile-based, mother-partner delivered intervention to improve BSE. The intervention is informed by a BSE framework, with two innovative enhancements: a) directly involving the partner as a conduit for increasing maternal self-efficacy, and b) tailored text message delivery, an approach designed to meet the specific time demands and changing needs of couples during the early postpartum period.

The current study is a randomized trial of a novel program, *Happy, Healthy, Loved (HHL*), designed to increase BSE through emphasizing partner support and actively addressing postpartum-specific stress management in mobile health program. It is expected that participants randomly assigned to the intervention, compared with those assigned to usual care, will report higher BSE, lower depressive symptoms, and higher perceived partner support at 6 weeks and 6 months postpartum.

## Methods

### Participants

Participants and their partners were recruited directly on the maternity floors of three midwestern hospitals. Participants were included if they were primiparous, at least 18 years old, living with a partner or spouse, and intending to breastfeed 6 weeks or more. Participants were excluded if they had infants in the Neonatal Intensive Care Unit, met criteria for a current depressive episode or suicide risk, were receiving antidepressant treatment or psychotherapy for depression, or did not speak, read and write English. Participant characteristics are summarized in Table 1.

**Table 1:** Participant characteristics

	Mothers		Partners		Total	
	N (*M*)	% (*SD*)	N (*M*)	% (*SD*)	N (*M*)	% (*SD*)
Age	(29.75)	(4.09)	(30.78)	(4.85)	(30.26)	(4.49)
American Indian	0	0	0	0	0	0
Asian/Asian-American	5	7.81	4	6.25	9	7.03
Native Hawaiian/Pacific Islander	0	0	0	0	0	0
Black/African American	1	1.56	5	7.81	6	4.69
White	55	85.94	49	76.56	104	81.25
Unknown/prefer not to respond	0	0	1	1.56	1	1.56
Hispanic/Latinx	5	7.81	5	7.81	10	7.81
Some high school	0	0	2	3.13	2	1.56
High school diploma/GED	8	12.50	6	9.38	14	10.94
Associate/technical degree	2	3.13	6	9.38	8	6.25
Bachelor’s degree	32	50.00	32	50.00	64	50.00
Graduate degree	22	34.38	18	28.13	40	31.25
Income < $25,000					6	4.69
$25—49,999					6	4.69
$50—74,999					22	17.19
$75—99,999					22	17.19
$100—124,999					26	20.31
$125 +					40	31.25
Prefer not to answer					6	4.69

Household income calculated per couple, not per individual

### Measures

Study data were collected and managed using REDCap electronic data capture tools hosted by the study hospital system. REDCap (Research Electronic Data Capture) is a secure, web-based software platform designed to support data capture for research studies [33, 34]. The following measures were used at one or more of the three data collection time points, in addition to basic demographic information.

#### Edinburgh postnatal depression scale [35]

The EPDS is a well-validated 10-item self-report depression screening tool in which high scores reflect more depressive symptoms within the past seven days. The EPDS has shown high sensitivity, specificity, and positive predictive power for postpartum depression when using a 10 + score cutoff [36, 37]. The EPDS has also been validated as an acceptable screening tool for men in the postpartum period [38].

#### Breastfeeding self-efficacy scale-short form [13]

The BSES-SF has demonstrated strong reliability and validity as well as high internal consistency (α = 0.91) [39], and has been validated for use with partners of breastfeeding women as well [40].

#### Postpartum partner support scale [36]

The PPSS is a 24-item self-report instrument to assess partner postpartum-specific support, with higher scores indicating higher levels of perceived support. This scale demonstrated reliability with mothers during the postpartum period (α = 0.96) and significantly distinguished between women with and without depressive symptoms at 8 weeks postpartum [36].

### Procedures

#### Enrollment

All study procedures were approved by the OhioHealth Institutional Review Board (IRB#1,296,284–1). Potential participants were identified and approached by the research team during the 2–3 day postpartum hospital stay. After determining interest and eligibility, mothers and their partners provided written informed consent.

Participants (both mothers and partners) completed pre-intervention surveys, then were randomized to either the intervention or control group according to a two-group randomization table uploaded to REDCap by the study statistician prior to enrollment.

Couples randomized to the intervention group then completed a brief interactive educational tablet program (Happy, Healthy, Loved) together, followed by 6 weeks of tailored text messages. Participants in the control group received usual care followed by 6 weeks of attentional control text messages about infant development. All patients received access to a lactation consultant during the hospital stay along with an optional in-person class, instructional video, and information packet about breastfeeding.

Participants were able to opt out of the text messages at any time in the 6 weeks. All program delivery was managed through contracted partnership with a company specializing in behavioral health platforms using HIPAA-compliant procedures. Development of the initial tablet-based prototype was designed in consultation with an instructional technologist at Denison University who contributed to a user-friendly tool.

**Fig. 1:**
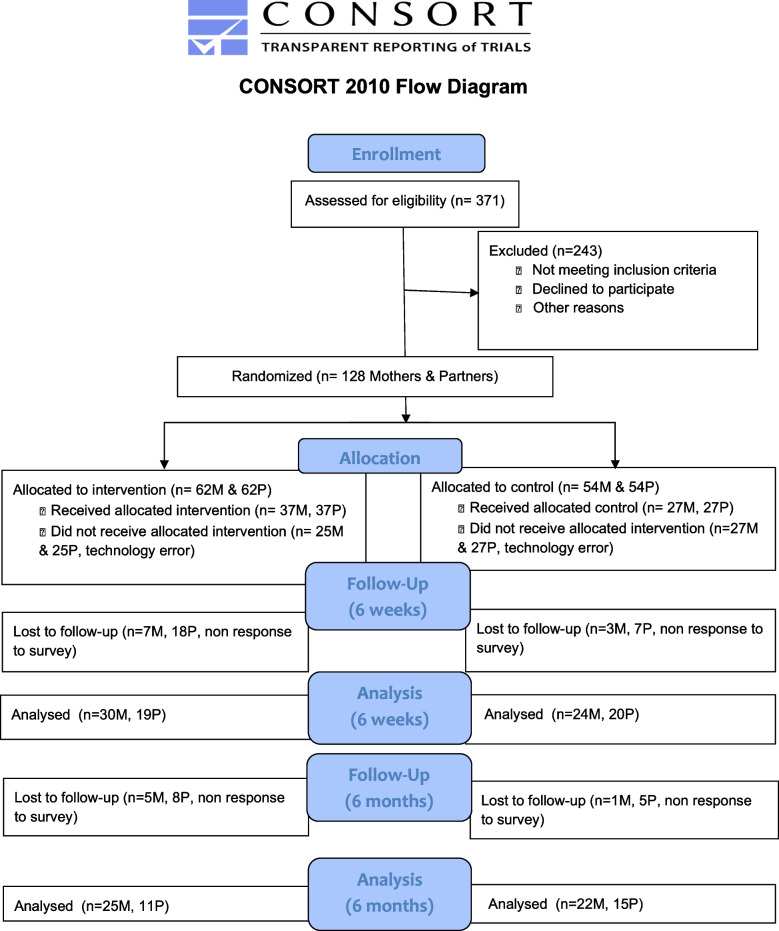
Study enrollment flow chart

Mothers were asked via text each week “How is baby eating this week?” with three text response options: 1 (all breastmilk), 2 (breastmilk and formula), or 3 (all formula). If mothers in the intervention reported that they were no longer breastfeeding, all remaining text messages emphasized coping and partner support only rather than breastfeeding, to minimize any guilt or distress a mother may feel for discontinuing breastfeeding. This occurred automatically within the program. A summary of participant recruitment and flow is found in Fig. 1.

#### Intervention

##### Happy, Healthy, Loved (Experimental Group)

The HHL program is comprised of three topical areas delivered by tablet to both parents during the postpartum hospital stay (see Table 2 ).

**Table 2:** Intervention goals and activities

	Goals	Example Activities
Modeling & Feedback	Viewing breastfeeding as learned skill	Other mothers’ modeling of managing challenges
	Reflecting on successes and growth	Feedback on breastfeeding success
Partner Support	Increase perceived support	Prompt partner to show active support
	Mutual support of self-care	Prompt both partners to support other’s self-care
Stress Coping	Behavioral activation	Prompt mother’s preferred rewarding activity
	Realistic expectations of self	Strategies for challenging negative thoughts

**Fig. 2:**
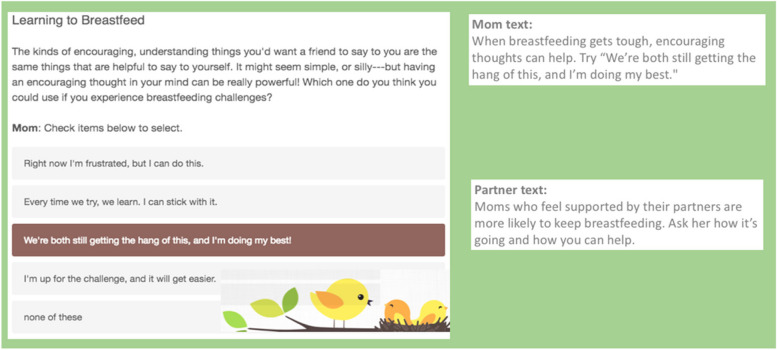
Intervention delivery sample of tailored texts

The content and delivery of the text-messages are closely mapped to the content provided in the tablet-based materials, providing reminders, resources, and encouragement associated with the three areas of education. Texts were matched to mothers’ response to questions in the educational module designed to identify her strengths and preferred coping strategies. For example, a mother who identified during the initial module that walking has been a positive coping strategy for managing stress and mood in the past would get a text specifically encouraging her to make time for a walk, with or without baby. The partner would receive a corresponding text suggesting that walking is helpful for the mother and encouraging the partner to provide support for this activity in one of two ways: offer to watch the baby while mother walks or go for a walk with the mother and baby. Example content is provided in Fig. 2.

### Statistical analysis

The intervention’s impact on the study’s primary outcomes were evaluated using intent-to-treat analysis with multiple imputation for missing follow-up cases. All results are presented using intent-to-treat sample with last case carried forward. All results were run using completed sample as well and any differences in outcomes are mentioned in text. Outcomes, adjusting for baseline variables, were analyzed using ANCOVA for continuous variables (self-efficacy, partner support, depressive symptoms). and Pearson Chi Square for categorical variables (exclusive breastfeeding).

## Results

Results of recruitment flow through 6 weeks and 6 month data collection (shown in Fig. 1) show that both survey timepoints were completed by 47 mothers and 26 partners. The majority of the sample was white, and most participants had completed a bachelor’s degree or higher. The household income level of the majority of participants was $75,000 or higher (Table 1).

### Primary outcomes

It was hypothesized that participants randomly assigned to the intervention would report higher BSE at 6 weeks and 6 months postpartum compared to those assigned to usual care. Descriptive statistics for all outcomes and groups are reported in Table 3.

**Table 3:** Breastfeeding and mood outcomes by group

	Intervention			Control			Total		
	*M (n)*	*SE (%)*	95% CI	*M (n)*	*SE (%)*	95% CI	M(n)	SE(%)	*p* < .05
Breastfeeding self-efficacy									
Baseline	2.74	0.14	2.46, 3.00	2.82	0.14	2.55, 3.10	2.77	.10	
6 week	3.38	0.16	3.06, 3.70	3.05	0.19	2.68, 3.42	3.24	.13	
6 month	3.69	0.15	3.39, 3.99	3.12	0.18	2.75, 3.46	3.44	.14	*
Breastfeeding exclusively	(11)	(42%)		(15)	(63%)		(26)	(52%)	
Partner infant support (PPSS)									
Baseline	3.79	0.25	3.71, 3.87	3.73	0.42	3.41, 3.83	3.73	.05	
6 week	3.63	0.06	3.52, 3.75	3.56	0.07	3.43, 3.70	3.60	.05	
6 month	3.59	0.06	3.46, 3.71	3.46	0.08	3.31, 3.61	3.53	.05	
Maternal depression									
Baseline	6.57	0.50	5.56, 7.53	7.70	0.75	6.20, 9.12	7.05	.44	
6 week	5.64	0.61	4.42, 6.86	6.64	0.72	5.21, 8.08	6.06	.50	
6 month	5.38	0.70	3.98, 6.78	6.92	0.08	5.29, 8.56	6.03	.56	
Partner depression									
Baseline	6.05	0.58	4.94, 7.20	6.70	0.71	5.25, 8.15	6.33	.44	
6 week	6.16	0.83	4.60, 7.82	6.81	0.95	5.12, 8.92	6.44	.60	
6 month	5.49	0.74	4.19, 7.05	7.11	0.88	5.48, 9.00	6.17	.55	

Breastfeeding exclusively represents percentage of participants reporting exclusive breastfeeding at 6 weeks

#### BSE

Results of an ANCOVA (baseline BSE covariate) do not support the hypothesized difference in self-efficacy at 6 weeks [*F*(1, 61) = 1.80, *p* = 0.19, η_p_^2^ = 0.03]; however, results show a significant effect of intervention group on 6 months BSE [*F*(1, 61) = 6.24, *p* = 0.02, η_p_^2^ = 0.09]. As expected, participants reported higher self-efficacy in the intervention group (*M* = 3.69, *SE* = 0.15, 95%CI = 3.39, 3.99) compared with the control group (*M* = 3.12, *SE* = 0.18, 95%CI = 2.75, 3.46).

#### 6-week breastfeeding exclusivity

Pearson Chi-Square assessing level of breastfeeding at 6 weeks postpartum (exclusively breastfeeding or formula introduced) x (intervention or control) shows no significant relationship between group and category of infant feeding [*X* ^2^(33) = 0.07, *p* = 0.80, Cramer’s V = 0.05].

### Secondary outcomes

#### Postpartum partner support

Results of an ANCOVA (PPSS baseline covariate) show no significant effect of intervention group on 6 week PPSS [*F*(1, 61) = 0.630, *p* = 0.430, η_p_^2^ = 0.01] or 6 month PPSS [*F*(1, 61) = 0.1.56, *p* = 0.22, η_p_^2^ = 0.03].

#### Maternal depressive symptoms (EPDS)

An ANCOVA with EPDS baseline as the only covariate showed no significant effect of intervention group on maternal depression at 6 weeks [*F*(1, 61) = 1.11, *p* = 0.30, η_p_^2^ = 0.02] or 6 months [F(1, 61 = 2.03, *p* = 0.16, η_p_^2^ = 0.03]. Similar, no differences were found in partner depression at 6 weeks [*F*(1, 57) = 0.00, *p* = 0.10] or 6 months [*F*(1, 57) = 0.00, *p* = 0.99].

## Discussion

The purpose of this study was to determine the initial efficacy of a tailored text message program for mothers and their partners to increase BSE and exclusive breastfeeding rates. In comparison with an attentional control group, no differences were found in the primary outcomes at 6 weeks postpartum; however, BSE was found to be significantly higher at the 6 month follow-up.

BSE has been determined to be malleable with intervention, as evidenced by a recent systematic review [41]. However, most existing interventions require more intense and direct provision of information, such as telephone counseling, multiple extended education sessions, or in-person workshops. The current intervention was intentionally designed to be scalable and easily delivered. This approach may not have enough depth of information to make the impact on breastfeeding that previous higher intensity interventions have established, or alternately, limited statistical power may contribute to the breastfeeding and self-efficacy outcomes. Understanding the lower limits of “dosage” for BSE interventions is an important step in designing interventions that maximize breastfeeding outcome impact while also maximizing the scalability and cost effectiveness of the program. Two recent interventions show promise in this mobile-delivered approach. Using social media support via WhatsApp for BSE, authors have found that a combination of pre-set educational messaging and personalized individual counseling (both based in WhatsApp) resulted in increased BSE among women in Turkey [42], while a similar WhatsApp approach was also found useful in Malaysia [43]. Determining best approaches for communicating support to mother-partner dyads will continue to be an important direction for future research. It should be noted that the current study sample is comprised of couples representing relatively high income and education levels, and little racial or ethnic diversity. The impact of these variables on the study intervention outcomes is unknown, and the acceptability and impact of similar interventions across more diverse demographic samples will be important to evaluate in the future.

The study findings have implications for self-efficacy theory as well. The current study finding that the text-based intervention was significantly effective at 6 months, but not at 6 weeks postpartum, suggests further study about the trajectory of BSE be conducted, particularly with first time mothers. Results in this study suggest that the intervention group continued to increase in self-efficacy over time, while control participants’ self-efficacy remained stable from 6 weeks to 6 months. A previous prospective study suggests that stability from this period is typical [44], adding further support to the potential impact of the program by increasing self-efficacy over time. Future exploration of the optimal timing and intensity of breastfeeding support over the first 6 months will be important for determining whether, for example, more intense support is needed during the first several weeks of breastfeeding to establish a beneficial self-efficacy trajectory.

Interestingly, while BSE did improve with the intervention group by 6 months postpartum, the intervention did not produce a difference in exclusive breastfeeding rates at either time point. While a relationship between BSE and exclusive breastfeeding is well established, the current results suggest that perhaps other structural or environmental factors (e.g., return to work) may be playing a role in breastfeeding exclusivity as well. It is possible that a longer follow-up time, such as a 1-year timepoint, would demonstrate different findings around exclusive breastfeeding.

The following limitations should be considered when interpreting the results of the study. First, COVID disruptions as well as an administrative error from the technology partner resulted in a smaller sample size than planned, limiting the statistical power to detect medium or small treatment effects. The recruitment across various levels of COVID restrictions meant that participants’ hospital and postpartum experiences of lockdown or return to work were varied, with the impact of these changes on the intervention unknown. Additionally, while passive engagement is a design strength of the study, it also is a limit, as we cannot know for certain how much the participants engaged in prompted behaviors delivered via text message. Partners engaged in follow-up surveys at lower rates than mothers, potentially suggesting lower engagement of partners, though this cannot be clearly determined.

Future breastfeeding-self-efficacy research should continue to explore the use of scalable and personalized technology, including the ability to adapt with greater nuance using machine learning and artificial intelligence tools. However, researchers should also pilot hybrid approaches that utilize adaptive text message and app-based data collection while also engaging mothers and partners in direct personal communication. Technology combined with personal communication may be especially helpful for overcoming the logistical challenges of including partners and other members of a support system in interventions.

## Conclusions

In conclusion, the current study tested the impact of a BSE intervention delivered to mothers and partners using tailored text message delivery. Results of the randomized trial show the intervention improved BSE at 6 months but not 6 weeks postpartum, indicating that text-based mother-partner interventions are a promising direction to continue exploring in postpartum health research.

## Data Availability

Current study deidentified data is available through the pre-registered trial at clinicaltrials.gov #NCT04578925 (registration date 2020–07-24).

## Footnotes

1 Our current study is inclusive of all gender and sexual orientation couples. The term father is used when referencing work cited in the literature as including exclusively fathers; partner is used when referencing our own study design. All birthing people in our study identify as women, so the term mothers will be used.
